# Virus tracking technologies and their applications in viral life cycle: research advances and future perspectives

**DOI:** 10.3389/fimmu.2023.1204730

**Published:** 2023-06-02

**Authors:** Di Liu, Li Pan, Huanjie Zhai, Hua-Ji Qiu, Yuan Sun

**Affiliations:** State Key Laboratory for Animal Disease Control and Prevention, Harbin Veterinary Research Institute, Chinese Academy of Agricultural Sciences, Harbin, China

**Keywords:** virus tracking, living-cell imaging, virus tracking technology, imaging microscopes, viral life cycle

## Abstract

Viruses are simple yet highly pathogenic microorganisms that parasitize within cells and pose serious threats to the health, economic development, and social stability of both humans and animals. Therefore, it is crucial to understand the dynamic mechanism of virus infection in hosts. One effective way to achieve this is through virus tracking technology, which utilizes fluorescence imaging to track the life processes of virus particles in living cells in real-time, providing a comprehensively and detailed spatiotemporal dynamic process and mechanism of virus infection. This paper provides a broad overview of virus tracking technology, including the selection of fluorescent labels and virus labeling components, the development of imaging microscopes, and its applications in various virus studies. Additionally, we discuss the possibilities and challenges of its future development, offering theoretical guidance and technical support for effective prevention and control of the viral disease outbreaks and epidemics.

## 1 Introduction

The use of fluorescence-labeling technologies allows for the labeling of a group of molecules being studied through covalent binding or physical adsorption of fluorescent materials. These technologies have the ability to reflect the information about research subjects. When applied to the study of viruses, these technologies can be used to track their replication, transmission, and other life activities within cells or hosts. Additionally, they can aid researchers in determining the expression levels of certain genes or proteins during biological processes, making them essential for screening antiviral drugs and studying viral infections and pathogenesis.

**Figure 1:**
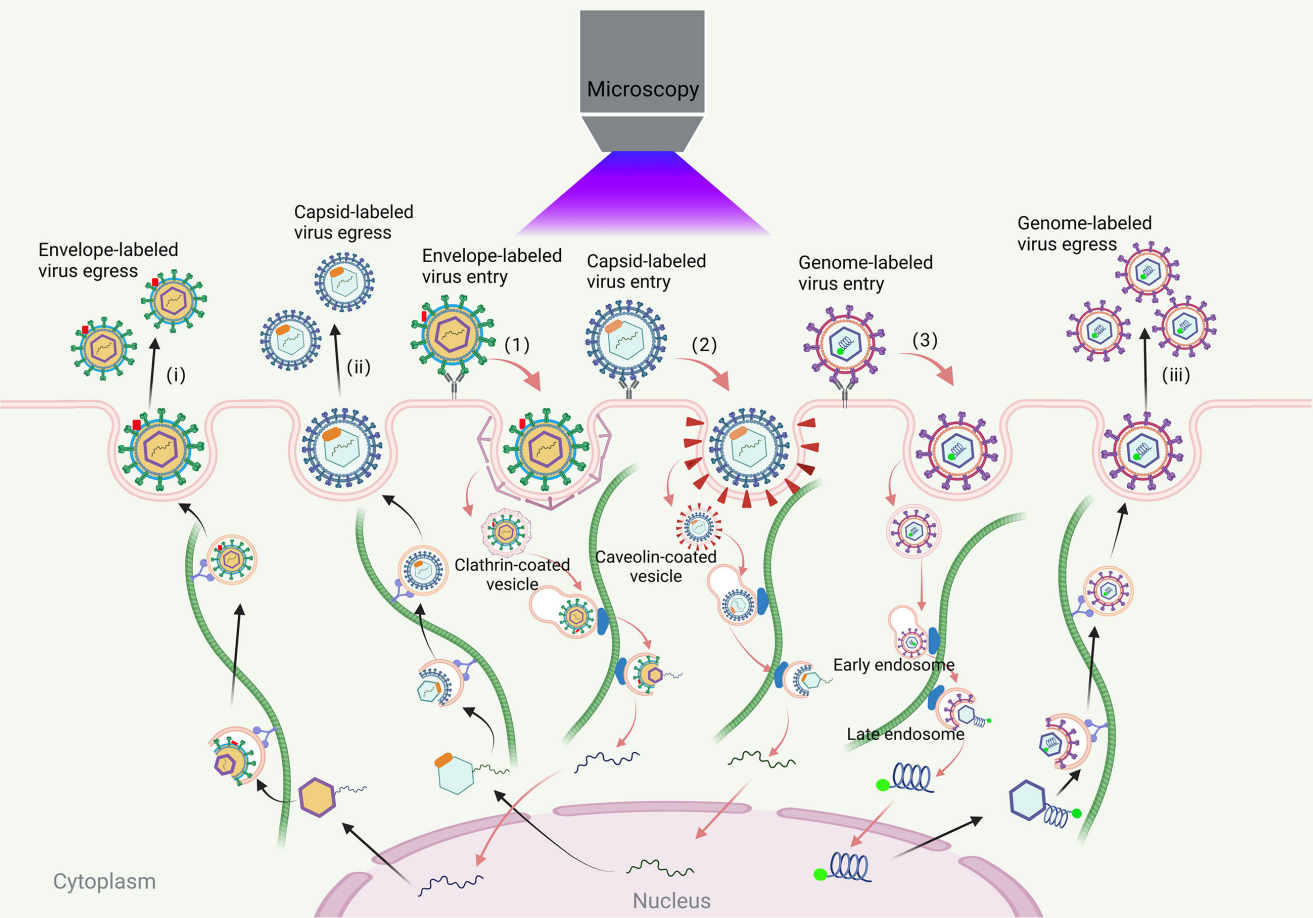
Visual tracking of the virus infection process. The process of viral infections in living cells includes virus entry, transport, assembly and release. The virus first binds to the receptors on the cell membrane. Most viruses enter the cell through clathrin, caveola-mediated endocytosis pathway and endocytosis pathway that does not rely on clathrin and caveola. The envelope-labeled virus (1) cannot be traced after entering the cell until the progeny virion is released outside the cell and they can be traced again (i); the coat-labeled virus (2) entering the cell, it can be transported along the cytoskeleton to the nucleus for fluorescence tracking until the virus is uncoated. The viral genome can be released into the cytoplasm or nucleus. The newly synthesized virus genome is then packaged into the capsid near the microtubule organizing center and it can be traced again until the progeny virion is released out of the cell (ii); genome-labeled viruses (3) can be traced throughout the life cycle of the virus (iii).

Viruses, which lack a cell structure and rely on host cells for replication, present a significant threat to the survival of humans and other animals (1). Their infection process involves complex interactions with various cell structures, including adsorption, internalization, uncoating, assembly, and release (2, 3). Traditional methods are insufficient for capturing the dynamic process and exploring the detailed mechanisms of virus infection. Virus tracking technologies allow researchers to track the infection process of virions and study the dynamic process of virus-cell interactions in real-time. This provides a more comprehensive understanding of the complicated dynamic process and infection mechanisms of viruses in time and space (**Figure 1**) (4–6).

In this review, we provide an overview of the selection strategies for labeling and discuss in details the fluorescence labeling strategies for different viral components. We also describe the imaging instruments for virus tracking and highlighted several applications of viral labeling technologies. Additionally, we discuss the future possibilities and challenges of the viral labeling technologies.

## 2 Selections of fluorescent labeling

### 2.1 Labeling methods and viral components

Labeling viruses poses practical limitations due to their dense structure, including challenges related to labeling approaches, tag sizes, and the precise positioning of labeled viruses. Careful consideration must be given to avoid compromising the functionality and replication levels of viruses during the labeling process. To address this, appropriate labeling methods and viral components must be selected, and thorough controls should be implemented to verify that the viruses retain their functionality and infectivity. Additionally, since virus assembly typically occurs within living cells, cellular labeling techniques are necessary to visualize the early stages of assembly. These limitations, distinct from labeling nanoparticles or other objects, make virus labeling particularly demanding and complex.

### 2.2 Viral size

The diameters of viruses typically range from 25 nm to 1 μm. To render viruses fluorescent, researchers have explored different techniques, one of which involves the insertion of a gene encoding fluorescent proteins (FPs) into the virus genome. By incorporating this gene, the virus becomes capable of producing the FP, resulting in the emission of fluorescent light. This method allows for direct visualization and tracking of the virus in host cells or tissues, enabling researchers to study viral replication, spread, and interactions with the host immune system or antiviral interventions. However, for larger viruses like vaccinia virus, traditional fluorescent labels such as green fluorescent protein (GFP) or red fluorescent protein (RFP) are preferred to avoid disrupting the virus’s structure and function. Conversely, influenza viruses, with an average diameter of only 100 nm, are difficult to label with traditional FPs due to their small size. For smaller viruses, chemical labels are often used to target lipids, proteins, or sugars in viruses (7, 8). These labels bind to specific functional molecules, but because the binding is non-specific, it is often unclear to know which molecules are labeled. However, new methods can add specific chemical labels to target molecules for more precise labeling (9, 10).

### 2.3 Fluorescence properties

The optical properties of fluorescent labels play a crucial role in visualizing viruses. Bright labels offer advantages in terms of higher temporal and spatial resolution, allowing for more detailed observations. Strong light stability is also essential as it enables longer tracking of viruses without significant signal degradation. Additionally, the spectral characteristics of fluorescent labels can be utilized to assess the quality of the labeling process. A high-quality fluorescent label should exhibit high brightness, facilitating efficient photon capture, and demonstrate excellent light stability to resist photobleaching caused by repeated excitation and de-excitation cycles. These optical properties are vital considerations for successful and reliable fluorescence labeling of viruses.

## 3 Fluorescence labeling strategies for different viral components

### 3.1 Membrane and capsid-based methods for visualization of viral infection

#### 3.1.1 FPs

**Figure 2:**
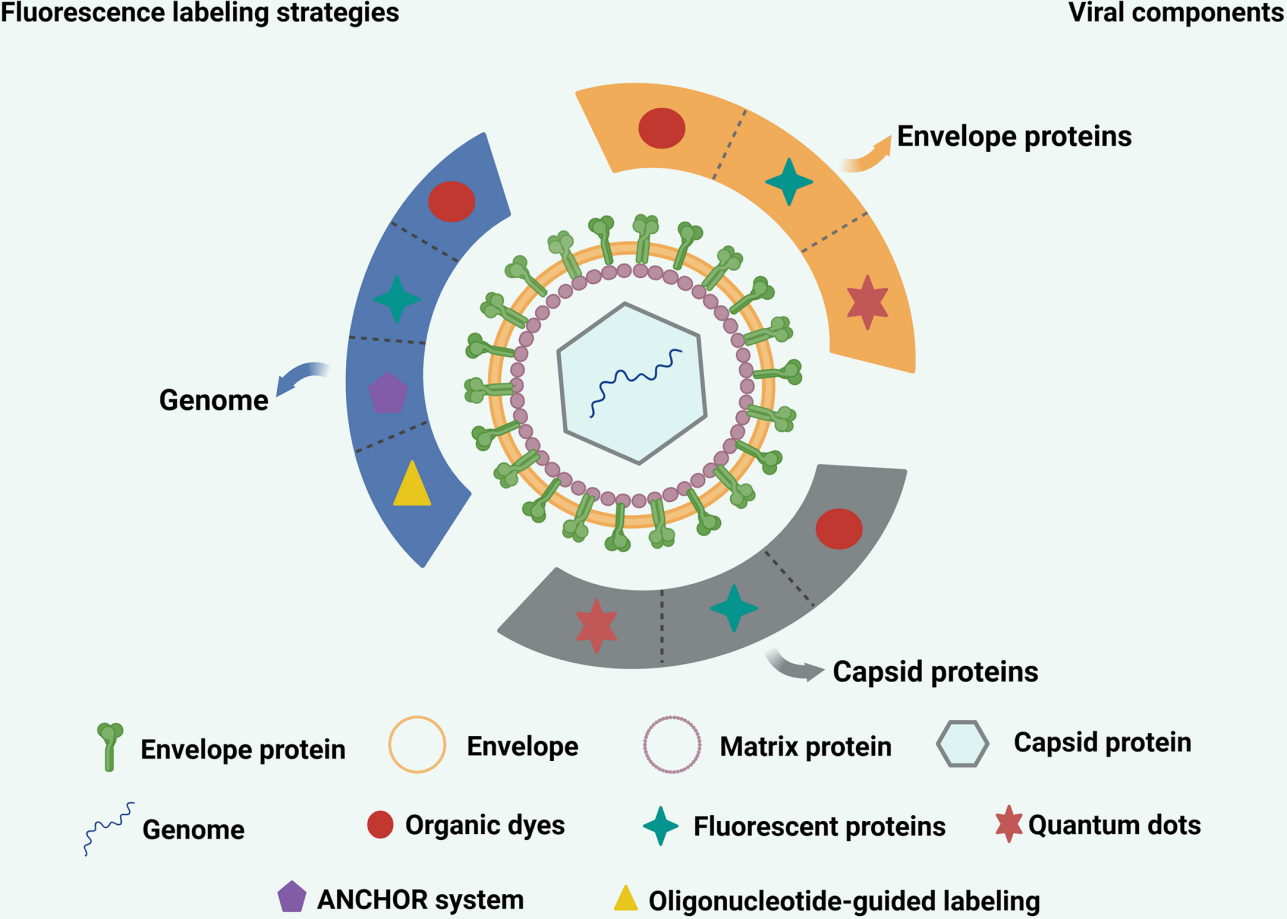
Commonly used strategies for labeling various viral components. The envelope proteins and capsid proteins of viruses can be labeled with fluorescent proteins, quantum dots and organic dyes. The genome of virus can be labeled with quantum dots, ANCHOR system, organic dyes and oligonucleotide-guided labeling.

Specific cellular and virus envelope and capsid proteins can be labeled using FPs through genetic engineering (**Figure 2**). Once the virus location is labeled, and it is confirmed that infectivity is not affected, there is no need to check whether labeling affects virus infectivity after each sample preparation. For these reasons, FPs are widely used in virology and have made significant contributions to the study of virus-cell interactions.

##### 3.1.1.1 GFP and GFP mutants

Green fluorescent protein (GFP) was originally discovered by Osamu Shimomura in *Aequorea victoria* in 1962, and was first expressed in *Escherichia coli* and *Caenorhabditis* in 1994 (11). GFP luminescence does not require other substrates or co-factors, making it suitable for monitoring gene expression and protein localization *in vivo* (12). Since then, researchers have conducted extensive studies on GFP and GFP-related research. The main advantage of GFP and GFP-like proteins is that their fluorescence is encoded in the sequence and can be retained when fused with other proteins. This breakthrough has enabled specific fluorescent labeling of proteins using simple molecular techniques in living cells. This discovery has also inspired researchers to create GFP mutants with better brightness, faster folding, less oligomerization, or different excitation and emission wavelengths (13, 14). One of the earliest enhanced variants is the enhanced green fluorescent protein (EGFP), which shows improved characteristics in mammalian cells. Wild-type GFP has inadequate brightness, folding, and excitation spectrum properties. Many GFP variants emit fluorescence in a range of 442 to 529 nm (15). In cell and molecular biology, GFP or GFP variants are often used as reporter genes to verify hypothesis. Through genetic engineering, the GFP gene can be transferred into the genomes of different species and continue to be expressed in offspring. GFP or GFP-like genes have been introduced and expressed in many species, including viruses, bacteria, fungi, fish (such as zebrafish), plants, and others. To obtain viruses labeled by GFP or GFP mutants, recombinant gene technology is used to fuse viral protein genes with GFP genes. The virus can be detected by fluorescence when recombinant cDNA cloning is transfected into living cells (16, 17).

##### 3.1.1.2 Phototransformable fluorescent proteins

Phototransformable fluorescent proteins (PtFPs) are capable of emitting fluorescence of different colors with the changes of excitation wavelength (18). These FPs have widely been used in super-resolution microscopy and have garnered significant attention (19, 20). Various PtFPs have been developed to meet the requirements for different colors and conversion modes, including photoactivatable, photoconvertible, and photoswitchable proteins, which have been employed to track the dynamics of cellular components in living cells (20–22). For example, Kaede, a GFP homolog discovered in 2002, can complete the transformation from green to red fluorescence upon stimulation by ultraviolet light (23). By tracking the apparent color changes of Kaede before and after light conversion, researchers can continuously monitor the dynamic changes of red fluorescence and then describe the dynamic characteristics of its labels. Super-resolution microscopy utilizes the photophysical characteristics of PtFPs to achieve high-resolution images. By sequentially activating and precisely localizing fluorophores, this technique can capture detailed images, facilitating the study of virus infection mechanisms. A dual-color super-resolution microscopy based on PtFPs has been utilized to visualize Gag assembly sites and HIV-1 Env proteins in both virus-producing and Env-expressing cells, as reported in a previous study (24).

##### 3.1.1.3 pH-sensitive fluorescent proteins

FPs have been subjected to numerous mutations, resulting in variants with various optical properties and environmental sensitivities that can change with the environmental pH (25, 26). pH affects the hydroxyphenyl group structure of the fluorescent protein chromophore, which can be either neutrally or negatively charged, and the conversion ratio of both cases is determined by the protonation equilibrium constant. Thus, pH-sensitive variants can be produced, allowing for the detection of pH changes. For example, pHluorin, a pH-sensitive GFP variant, consistently exhibits high fluorescence under neutral condition and significantly reduced fluorescence under acidic conditions. Therefore, these FPs offer unique advantages in studying the transport mechanisms of endocytosis and exocytosis in living cells (27, 28). As many viruses use the endocytosis pathway to release their genomes, targeting pH-sensitive FPs to viruses can be employed to analyze virus-endosomal fusion in real-time within living cells. Therefore, pH-sensitive FPs are useful fluorophores for studying viral infection mechanisms (29–31).

#### 3.1.2 Peptide tag-mediated labeling

Fluorescent proteins, as genetically encoded labels, have high specificity but relatively low brightness and poor light stability (32). Organic dyes and nanoparticles usually have better photophysical properties but cannot achieve site-specific labeling in living cells. To combine the advantages of both, a class of proteins have been developed that can catalyze the attachment of fluorescent ligands in living cells (33). These proteins are non-fluorescent, but fluorescent ligands can label them, resulting in more stable, brighter, and color-tunable labels with properties similar to FPs, such as environmental sensitivity, but with better photophysical properties (34–36). The labeling of imaging probes is controllable in space and time, and the color of the target protein can be selected according to the need of the tests (37).

Self-labeled fusion tags have a recognition domain that can provide fluorescent ligands to attach specifically to the target protein in living cells, allowing the target protein to bind to the self-labeled fusion peptide or protein sequence. In living cells, the protein is expressed, and specific fluorescent ligands are added for protein labeling. The short sequence of self-labeled fusion peptides and proteins makes it easy to create with various fluorescent ligands (10). The His tag consists of at least six histidine residues, which have high affinity and selectivity for Ni^2+^ and is often used to purify the protein. His tag has been used for live cell imaging under the development of Ni-NTA-based fluorescent probes (38–40). For instance, Ni-NTA functional quantum dots (QDs) interspersed with PEG have been developed to label the prion expressed on cell surfaces (40). In addition, Huang et al. developed a Ni-NTA-based virus labeling strategy by coupling specific polypeptides containing His tag with the virus surface, which is non-invasive and can label the virus envelope (41). The tetracycline (TC) sequence, with only six amino acids (most commonly CCPGCC), is one of the shortest peptide labels available. It can bind to green fluorescence (FlAsH) or red fluorescence (ReAsH), allowing specific proteins to fluoresce. For example, recombinant vesicular stomatitis virus (VSV) can be labeled with double fluorescence by fusing M protein with TC labels and P protein with EGFP. Time-sequence images confirmed the adsorption of VSV on the cell membrane and showed that the entry and uncoating of VSV in infected cells took place with a half-life after the virus adsorption (42). However, the interaction with other thiol-containing proteins can result in high background signal and require a long wash before imaging.

#### 3.1.3 Lipophilic fluorescent dyes

Organic fluorescent dyes, which are small (<1 kDa), have proven to be essential for labeling biological systems with fluorescence. They are often excited with high energy and short wavelength due to their single-photon excitation process, while the emitted light has low energy and long wavelength characteristics (43). The advantages of organic dyes include high selectivity, sensitivity, and fluorescence quantum yield, making them widely used for tracing viruses (7, 8).

Lipophilic fluorescent dyes possess the ability to stain cell membranes and other lipid-soluble biological structures. Enveloped viruses acquire their lipid membrane from host cell or organelle membranes. Through hydrophobic-photophilic interactions, lipophilic dyes can incorporate into the viral envelope. At high concentrations, self-quenching occurs when these dyes are integrated into virus particles. However, during virus fusion with the cell membrane under low pH conditions, the concentration decreases, leading to dequenching of the lipophilic dyes and an increase in fluorescence signals. Consequently, lipophilic dyes can be utilized to detect the release of the virus genome, with enhanced fluorescence indicating virus-plasma membrane or virus-endosome fusion (44, 45).

For instance, influenza viruses infect cells through the endocytosis pathway, which requires the combination of the viral envelope and the endometrium to transmit the viral genetic material to the cytoplasm. The R110-labeled influenza viruses were used to study real-time hemifusion and its pore formation on the double layer of lipids. Hemifusion occurs in the rapid brightening of the individual, which is caused by the quenching of R110 (44). Long-chain dialkyl carbocyanines, such as DiO, DiI, and DiD, are extraordinarily lipophilic due to their unique structure and can be combined with lipid-soluble biological structures. At a certain concentration, the lipid membrane can be completely stained. After being excited, the long-chain dialkyl carbocyanines have a high quenching coefficient and excitation lifetimes, which play a significant role in the tracer of enveloped viruses. Deep-red lipophilic dyes, such as DiD, with high fluorescence signal, are not easy to quench and have low self-fluorescence interference. They have been widely used to trace dengue virus (DENV) (46, 47), hepatitis C virus (HCV), avian sarcoma leukemia virus (ASLV) (48), and human immunodeficiency virus (HIV) (49). Green lipophilic dyes, such as DiO, and orange lipophilic dyes, such as DiI, have also been used to monitor the infection behavior of viruses, such as Ebola virus (EBOV), vesicular stomatitis virus (VSV), hepatitis B virus (HBV) and HIV (50–52).

### 3.2 Nucleic-acid-based methods for visualization of viral infection

#### 3.2.1 Intercalating dyes

Whole virus particles package the viral genome, making it difficult for dyes to bind to the genome. However, some intercalating dyes can penetrate the external components of virus particles and label the virus genome (**Figure 2**). One such dye is ribogreen, which is an intercalating dye with slight fluorescence and negligible absorption. This dye is fluorescent, meaning that its fluorescence intensity is amplified by several orders of magnitude when it binds to nucleic acids. Ribogreen has been used to detect and quantify RNA and DNA. When ribogreen is incubated with human rhinovirus (HRV), it comes into contact with the viral genome and binds during “capsid breathing”. Other intercalating dyes, such as YOYO-1 and SYTOX orange (SxO), are almost non-fluorescent in solution and have a high affinity for double-stranded DNA, resulting in a 1,000-fold increase in fluorescence intensity. Stained DNA molecules are easy to detect at the single-molecule level due to the increase in signal-to-noise ratio, making these intercalating dyes essential tools for single-molecule imaging. However, intercalating dyes can distort the DNA structure, which can not only inhibit protein-DNA interaction but also lead to an increase in length parameters. These parameters strongly depend on the concentration of intercalating dyes, and reducing dye concentration can reduce the distortion effect on the DNA structure. However, using low-concentration dyes can make the fluorescence signal difficult to detect or quickly fade. Therefore, when conducting single-molecule experiments, intercalating dyes should be handled with care (53). SYTO 82 is an orange fluorescent nucleic acid binding dye that has successfully labeled the viral genome of RNA viruses, including poliovirus (PV) and influenza viruses (54–56).

#### 3.2.2 ANCHOR system

The ANCHOR system is a binary system derived from the chromosome separation mechanism of the bacteria ParABS. In its natural form, the ParABS system consists of a short, non-repeating target DNA sequence containing a limited number of ParS nucleation sites, to which ParB proteins bind and then spread to adjacent DNA *via* the mechanism of protein-protein interaction. The third component of the system is the ATPase, which involves the final step of bacterial chromosome or plasmid separation. In its engineering form, called ANCHOR, the shortened ANCH sequence, including palindromic ParS nucleation sites, is specifically bound to ParB proteins. If ParB proteins fuse with FPs, their accumulation on the ANCH target sequence and spread to adjacent sequences will form fluorescence focuses being easy to detect, enabling the identification of ANCHOR-labeled DNA sites (57, 58). The ANCHOR system has been successfully used to analyze the infection and replication process of various virus, such as human cytomegalovirus (HCMV) (59), vaccinia virus (60), and baculovirus, and the myxoma virus based on the ANCHOR system can be used to evaluate antiviral drugs (61, 62). The ANCHOR system has been proven not to disrupt the structure and function of chromatin, despite the presence of up to 500 ParB proteins on and around the ANCH sequence (63).

#### 3.2.3 Oligonucleotide-guided labeling

Oligonucleotide-guided labeling is a method of analyzing target DNA or RNA through various forms of hybridization with the target nucleic acid molecule. Organic dyes or QD-labeled oligonucleotides have been designed and can be transfected into virus-infected host cells to bind to the viral genome. During the virus assembly process, oligonucleotide-labeled fluorophores can be assembled into the virus. For example, QD-labeled guide RNAs containing the packaging signal sequence of the virus genome can encapsulate functional QDs into the capsid of VSV glycoprotein pseudo-moderate virus (PTLV) in living cells (64).

Labeling the virus oligonucleotide enables tracking of the virus genome and the replication location. Fluorescence *in situ* hybridization (FISH) is a technology that utilizes complementary base pairing to bind a specific DNA sequence to the target sequence within cells. This allows for the direct visualization of specific nucleic acids within cells, facilitating the observation of virus-related sequences and their localization.

The probe used in FISH is fluorescent, and under appropriate excitation light, the hybrid probe and the target DNA can be clearly observed under a fluorescence microscope. Traditional fluorescent dyes used in FISH detection have a relatively wide emission spectral range and are prone to photobleaching, which significantly limits their application in signal amplification. However, as semiconductor nanocrystallines, QDs are mainly composed of group II-VI or group III-V inorganic elements and can produce fluorescence when excited by light (65). Combining FISH with QDs technology has enabled the detection of human papillomavirus (HPV) infection in patients with cervical squamous carcinoma. In addition, by using multiple fluorescent probes for the same molecule, FISH can be combined with flow cytometry to visualize the process of virus infecting cells. For instance, the Epstein-Barr virus (EBV)-encoded small RNAs (EBERs), which are noncoding RNAs abundantly expressed in the latently EBV-infected cells (66). It can be hybridized to the intracellular target mRNA *via* a FISH probe. Then, a specific antibody is used to label the cluster of differentiation (CD) (67), enabling flow cytometry to identify cell types of EBV infection and provide better diagnostic advice as well as new insights into the pathogenesis of EBV.

## 4 Development of optical instrumentation for virus tracking

### 4.1 Wide-field fluorescence microscopy

Wide-field fluorescence microscopy is a technique to excite fluorophores in samples using specific wavelength excitation and to receive fluorescence signal imaging through eyepieces or cameras. When the fluorescent molecule is irradiated by excitation light at a specific wavelength, extranuclear electrons in the ground state absorb excitation photon energy and jump to the excited state. Some extranuclear electrons of fluorescent molecules at the high energy level first jump to the metastable energy level in a non-radiation transition and then to the ground state as a radiation transition. Photons corresponding to the energy wavelength will be emitted during the radiant transition, forming fluorescent signal light, which can be captured by eyepieces or cameras for fluorescence imaging.

In the wide-field fluorescence microscopy, the large field of view is entirely illuminated by excited light, and the camera can quickly and easily capture the fluorescence signals of a single sample (68). Objectives with an NA > 1.2 are typically used to obtain a large number of signals, along with sharp fluorescence filters with high transmission (>80%). For detection, fast frame transmission and high quantum yield cameras are essential for imaging living cells of viruses. The fluorescence imaging of the sample can be observed directly with the naked eye or captured by a camera. The two most commonly used photoelectric sensors are Charge-Coupled Devices (CCD) and Complementary Metal Oxide Semiconductors (CMOS), depending on different frame rates, noise, and sensitivity (69, 70). Wide-field fluorescence microscopy has the advantages of fast imaging speed, high resolution, simple structure, and relatively low cost. However, for high-background experiments, such as investigating the HIV assembly of Gag proteins marked by FP, the defocus signal cannot distinguish the depth of the sample that produces fluorescence, making wide-field fluorescence microscopy unsuitable for 3-D sample fluorescence imaging. Therefore, the wide-field microscopy is more suitable for 2-D imaging (71, 72).

### 4.2 Confocal microscopy

Minsky, a researcher at Harvard University, designed the first confocal microscope in 1955 while trying to image the connections between intermediate neurons. He realized that he could significantly improve the resolution by illuminating a small part of the sample and scanning point by point, which reduced the amplitude of light scattering. The basic principle of a confocal microscope is to use point lighting and pinholes in front of detectors to eliminate out-focal signals. The laser beam emitted by the light source of the confocal system passes through the lighting pinhole and becomes a point light source P. After the point light source P enters the imaging system of the high-numeric aperture microscope through the beam splitter, an image point P is formed in some transparent samples under test. Scattering and reflected light, and other lights emitted from the image point P, pass through the objective and spectroscope in the opposite direction and are incident on the single-point detector. The single-point sensor consists of a detector and a pinhole placed in front of the detector, which can reduce the effective area of the detector. Detectors generally use photomultiplier tubes or avalanche photodiodes, and the computer finally processes the electrical signals received by the sensor.

Compared with the wide-field fluorescence microscope, the confocal microscope excites only one point in the focal plane at a time. The adjacent fluorescent molecules are not excited and are unlikely to produce stray fluorescence (73–76). In addition, the resolution of the confocal microscope is higher than that of wide-field fluorescence microscope. In the confocal microscope imaging system, a point light source is used to illuminate the sample, and the point detector collects the light carrying the sample information. Finally, the three-dimensional information of the whole sample is obtained by transverse and longitudinal scanning technology. Spinning-disk confocal microscopy (SDCM) has become an indispensable tool for studying the dynamic events of biomolecules in living cells. Additionally, the combination of SDCM and fast piezoelectric Z scanning devices allows the recording of quick 3-D images and has been used for 3-D single virus tracking in living cells (77, 78).

### 4.3 Super-resolution microscopy

Over the years, the resolution of wide-field and confocal microscopy has been limited by the Abbe/Rayleigh diffraction limit, which cannot distinguish structures below 200 nm. In recent years, with the emergence of new fluorescent molecular probes and the improvement of imaging methods, researchers have developed various super-resolution fluorescence microscopies. These technologies have been greatly improved the resolution of optical imaging, achieving accuracy comparable to that of electron microscopy, and enabling the visualization of nanoscale proteins (10 to 50 nm) in living cells. An essential area of super-resolution imaging applications is living cell imaging, where techniques such as saturated structured illumination microscopy (SSIM), photoactivated localization microscopy (PALM), and stimulated emission depletion (STED) microscopy (79–81). For example, Chojnaki et al. used STED microscopy to demonstrate the capping of Env on mature HIV surfaces, indicating that the virus particle structure is ready to bind to target cells (82). Moreover, nanometer measurements of separated particles that have undergone virus entry and nuclear input show that the sequential change of particle size corresponds to a series of structural rearrangements related to virus entry (83, 84).

### 4.4 Two-photon fluorescence microscopy

Two-photon fluorescence microscopy is a novel imaging technology that combines laser scanning confocal microscopy and two-photon excitation technology. The basic principle of two-photon excitation is that, under high photon density, fluorescent molecules can absorb two long-wavelength photons simultaneously and then emit a shorter-wavelength photon after a brief excitation state. The resulting effect is that the fluorescence is excited by a photon with half the wavelength of the traditional single photon excitation method. Two-photon excitation requires a high photon density, which is achieved using a high-energy mode-locking pulse laser to avoid damaging cells. The laser used in two-photon microscope emits high peak energy and average deficient energy, with a frequency range of 80 to 100 megahertz (MHz).

When using a high numerical objective lens to focus the pulsed laser photon, the photon density at the focus of the objective lens is the highest. Two-photon excitation occurs only in the focus of the objective lens, so two-photon microscope does not need to confocal pinholes, improving fluorescence detection efficiency. Traditional confocal microscopes are prone to phototoxicity due to the small size of the confocal pinholes required to obtain high-resolution images. The small aperture blocks a significant proportion of fluorescence emitted from the sample, including the fluorescence emitted from the focal plane. Consequently, the excitation light must be strong enough to obtain a sufficient signal-to-noise ratio. However, high-intensity lasers can quickly bleach the fluorescent dye during the continuous scanning, and the fluorescence signal will become weaker and weaker as the scanning process continues. Additionally, many fluorescent dye molecules produce cytotoxins such as singlet oxygen or free radicals under laser irradiation, so the scanning time and the excitation light power density should be limited in the experiments to maintain the sample’s activity.

Under a two-photon fluorescence microscope, fluorescent dye molecules can absorb two low-energy photons simultaneously (the interval between two photons reaching the fluorescent molecule is less than one femtosecond), and the excitation effect is equivalent to absorbing a high-energy photon with a 1/2 wavelength. For example, absorbing two red wavelength photons is equivalent to one molecule absorbing ultraviolet. Long-wavelength photons are not easily absorbed by cells, reducing the phototoxicity to living cells and light bleaching, thus serving as an effective alternative to ultraviolet excitation and avoiding damage to the sample (85, 86).

## 5 Applications of DNA virus-labeling technologies in viral replication mechanisms

### 5.1 Virus adsorption

The adsorption of most viruses can be classified into two stages: non-specific adsorption and specific adsorption: Non-specific adsorption occurs when viruses come into contact with host cells through electrostatic forces. Specific adsorption, on the other hand, refers to the specific recognition and binding of viral surface proteins (antigens) to the corresponding receptors on the host cell membrane. The binding of viruses to receptors triggers downstream signals and initiates the internalization of viruses (2, 87). However, the detailed mechanism of how virus binding induces receptor aggregation or signaling is not yet fully understood, as the interactions between viruses and receptors occur in an instant and are challenging to analyze using conventional biological methods (88).

Recent studies have highlighted the critical role of filopodia during virus infection (89–92). Filopodia are thin, finger-like protrusions that extend from the surface of some cells. Researchers tracking a single mouse leukemia virus (MLV) found that the virus initially attached to filopodia and rapidly moved transversely along the filopodia, relying on action protein-dependent movement, before entering the cell body (89). Similarly, by monitoring the movement of horizontal HPV on the cell surface, four HPV migration patterns were identified, and it was found that HPV infection was facilitated by directional movement along actin protrusions, such as filopodia or retracted fibers (90).

### 5.2 Virus internalization

After the virus binds to the receptor on the cell surface, there are two main strategies for virus internalization: endocytosis-independent and endocytosis-dependent internalization. The first is that the virus binds to the receptor on the cell’s surface and then fusion directly with the plasma membrane into the cell, such as the herpes simplex virus (HSV) (93, 94). In the past, researchers mainly used traditional technologies such as transmission electron microscopy and inhibition tests to study the endocytosis-dependent internalization of viruses. However, a deep understanding of the internalization entry mechanism and dynamic process of most viruses is needed. Real-time virus tracking technology promotes our understanding of the virus entry mechanism, especially in live cell imaging with multicolor combinations (95). For example, by tracking the behavior of infected influenza viruses in living cells, the entry of influenza viruses follows two different pathways: clathrin-mediated pathways and clathrin- and caveola-independent pathways (87, 96). Meanwhile, tracking the pits of viruses and clathrin envelopes shows that most viruses promote the formation of clathrin packets around the virus from scratch and use clathrin-mediated virus internalization, and the rest of the viruses enter cells *via* the clathrin- and caveola-independent pathways, which have a similar efficient pathway.

### 5.3 Virus uncoating

After the virus enters the cell, it is necessary to release its genome from the package of the capsid to a specific site or nucleus of the cytoplasm before it can start subsequent replication. As the fluorescent dyes, SYTO 82 and CypHer5 were used to label the RNA and capsid of poliovirus (PV), respectively (55). The genome release of the virus was observed in real-time under a fluorescence microscope. It was found that the RNA release of PV only occurred in the vesicles near the cell membrane after the viral particles entered the cell. This study has solved the long-standing debate about the RNA release site of PV. Qin et al. (97) used QDs-conjugated viral ribonucleoprotein complexes (vRNPs) of influenza viruses to track the shelling process of a single virus. It was found that eight vRNPs of the virus were released from the late endosome to the cytoplasm alone rather than in groups during the shelling process. Each viral RNA (vRNA) entered the nucleus in a typical three-stage motion mode and then got to its replication sites in two different diffusion modes.

### 5.4 Virus transport

Many viruses enter the cell through the endocytosis pathway, finding that they are in intracellular vesicles and are transported to specific sites in the cell for genome release and replication. This mechanism often uses the cytoskeleton for intracellular transport, including myosin, dynamic protein, and driving protein. Therefore, virus transport is a complex and multi-step process, and viruses usually follow several pathways in the cytoplasm. Virus tracking technology can monitor the behavior of each virus-infected cell in real-time, thus providing new mechanism insights for virus infection (49). For example, influenza viruses follow a five-stage process of intracellular transportation: the virus is initially attached to the surface of the cell, moves slowly along the actin filaments around the cell and to the nucleus in a microtubule-dependent manner, and then interacts and fuses with the late endosome to release the genome (98). In addition, influenza viruses may be affected by microtubule structures in the cytoplasm. In living cells, the microtubule structure affects the fate of a single virus (99). Some of the findings show that during the virus transport, retrograde motor protein, myosin VI, and kinetic protein are responsible for the seamless virus transport from microfilaments to microtubules (100). The 3D tracer of influenza viruses shows that the different transmission behaviors of the virus are related to the early and late endosomes that their transformation appears in the perinuclear region (101).

### 5.5 Assembly and egress of viruses

After virus genome replication and protein synthesis, the viral genome is assembled with proteins to form well-structured and infectious progeny virions. The progeny virions are released from the host cell in many ways, such as vomiting, pyrolysis, or germination. Studying the dynamic mechanism of virus assembly and release are more challenging than that of virus invasion and virus transmission, because it is difficult to label the newly synthesized viral components with fluorescent labels. For example, Liu et al. developed a recombinant pseudorabies virus (PRV) based on the Halo Tag fixed-labeled virus capsids and combined with a variety of the Halo Tag protein fluorescent ligands to study the virus entry, intracellular transportation of parental viruses, as well as the assembly of progeny virions capsids, and the diffusion movement of the nucleus and cytoplasm, to complete the tracking of the life cycle of PRV (102).

### 5.6 Cell-to-cell transmission of viruses

After the progeny virions are release, they continue infecting new host cells. One mechanism of this infection is the virus transmission between cells. The progeny virions invade the new host cell through direct cell-to-cell contact, which may be 100 to 1000 times more efficient than cell-free transmission (103). Surprisingly, this transmission process is less sensitive to neutralizing external environments such as antibodies and antiviral drugs. Three typical physical connections between cells-membrane nanotubes, filamentous pseudopods, and virological synapses-significantly increase the spread of many viruses between cells (92, 104–108). Through virus tracking technology, the virus transmission between cells can be visualized, and its potential mechanism can be further revealed through dynamic processes.

## 6 Conclusions and perspectives

Virus tracking technologies are crucial for studying the virus replication process and understanding the mechanisms involved in the virus life cycle, including virus adsorption and internalization, transportation, genome delivery, assembly, and egress. The development and application of virus labeling technology require interdisciplinary knowledge from biology, virology, chemistry, and physics. Despite the rapid progress made in virus labeling technology, several challenges need to be addressed in the future to improve the accuracy and efficacy of virus labeling and to expand its range of applications.

Firstly, most virus particles are small, and fluorescent labels may affect the virus’s infectivity to varying degrees. To minimize this impact, a limited number of fluorescent labels should be used to attach to the virus composition. Additionally, conventional genetic manipulation methods can be used to label viral components with FPs, but the operation is complicated, the fluorescent intensity is weak, and the size of the FPs is large, which can also affect virus activity. Furthermore, continuously monitoring the virus infection process under microscope is challenging. The fluorescent labels used for virus tracing must have excellent performance, including strong fluorescence signaling, non-toxicity, anti-quenching, high stability, small size, ease of operation, and no impact on virus infection activity. For example, QDs have high fluorescence intensity and light bleach resistance, but they also have some limitations, such as large size, non-specificity, and potential impact on virus infection (10, 109–111).

Secondly, an urgent problem that needs to be addressed is continuously monitoring the entire infection process of fluorescence-labeling viruses at high resolution. While different components of the virus such as the viral envelope and capsid can be labeled with fluorescence, labeling nucleic acids remains a challenge (10), which limits the ability to trace the entire virus infection process. Multicolor labeling can facilitate the observation of the continuous infection process of the virus. However, efficient labeling of different viral components such as membranes, capsid, and nucleic acids, as well as effective distinction between parental and progeny viruses, require further exploration.

Finally, the virus infection process involves complex interaction between viral proteins and host cells. Given that some of these interactions occur between a small number of viruses and cell molecules, higher temporal and spatial resolutions are often required to efficiently monitor the relevant processes. However, fluorescence microscopy also has some limitations, such as imaging speed and resolution trade-offs, and cumbersome instrument operation. Confocal microscopy is a popular technique for living cell imaging, but it is limited by its resolution and cannot image structures smaller than 200 nm (112). 3-D imaging can provide more information than 2-D imaging, but it relies on high spatiotemporal resolution imaging instruments. Cutting-edge technologies are now combining physics, mathematics, and computers to optimize and develop image analysis algorithms, including advanced methods such as computer vision and artificial intelligence machine learning. By improving the automation and personalization of algorithms, optimized image processing technology can help researchers find and summarize the laws underlying many virus trajectories and deeply explore the mechanisms underlying viral infections.

## Author contributions

DL wrote the manuscript. LP, HZ, YS, and H-JQ revised this manuscript. All authors contributed to the article and approved the submitted version.
